# Supplementary material for: Climate Crisis and Mental Well-Being: Nature Relatedness, Meaning in Life, and Gender Differences in a Jewish Australian Study
Source: Behav Sci (Basel). 2025 Aug 1;15(8):1045. doi: 10.3390/bs15081045 (PMC12383100; doi:10.3390/bs15081045)
Supplement: Supplementary file 1 [file behavsci-15-01045-s001.zip › behavsci-3738891-supplementary.pdf]

Supplementary 1.

Table S1. Means and standard deviations of NR and MLQ measures by dichotomous background variables

Variable	*n*	Nature relatedness		MLQ Search		MLQ Presence	
		M	*SD*	*M*	*SD*	*M*	*SD*
Gender							
Men	14	3.59	0.80	20.36	8.23	26.93	4.01
Women	21	4.07	0.61	20.86	7.72	25.00	6.82
Birthplace							
Australia	21	3.90	0.70	21.76	6.58	24.90	6.65
Other	14	3.84	0.78	19.00	9.38	27.07	4.36
Family status							
In a relationship	25	3.78	0.72	19.92	8.25	25.80	5.06[i]
Not in a relationship	10	4.11	0.70	22.50	6.62	25.70	7.86
Economic status							
Doing well	16	4.08	0.59	22.81	7.52	26.38	7.07
Doing very well	19	3.70	0.79	18.84	7.78	25.26	4.77

Note. MLQ = Meaning in Life Questionnaire.
